# Challenges in conducting clinical trials in nephrology: conclusions from a Kidney Disease—Improving Global Outcomes (KDIGO) Controversies Conference

**DOI:** 10.1016/j.kint.2017.04.019

**Published:** 2017-08

**Authors:** Colin Baigent, William G. Herrington, Josef Coresh, Martin J. Landray, Adeera Levin, Vlado Perkovic, Marc A. Pfeffer, Peter Rossing, Michael Walsh, Christoph Wanner, David C. Wheeler, Wolfgang C. Winkelmayer, John J.V. McMurray, KDIGO Controversies Conference on Challenges in the Conduct of Clinical Trials in Nephrology Conference Participants, Ali Abu-Alfa, Patrick Archdeacon, Geoffrey A. Block, Fergus J. Caskey, Alfred K. Cheung, Bruce Cooper, Jonathan C. Craig, Laura M. Dember, Garabed Eknoyan, Ron T. Gansevoort, John S. Gill, Barbara Gillespie, Tom Greene, David C. Harris, Richard Haynes, Brenda R. Hemmelgarn, Charles A. Herzog, Thomas F. Hiemstra, Lesley A. Inker, Meg J. Jardine, Vivekanand Jha, Lixin Jiang, Kirsten L. Johansen, Reshma Kewalramani, Hiddo J. Lambers Heerspink, Martin Lefkowitz, Charmaine E. Lok, Fiona Loud, Romaldas Mačiulaitis, Dugan W. Maddux, Franklin W. Maddux, Magdalena Madero, Segundo Mariz, Michael Mauer, Joseph V. Nally, Masaomi Nangaku, Ikechi G. Okpechi, Patrick S. Parfrey, Roberto Pecoits-Filho, Brian J.G. Pereira, Michael V. Rocco, Patrick Rossignol, Franz Schaefer, Francesca Tentori, Aliza Thompson, Marcello Tonelli, Allison Tong, Robert D. Toto, Katherine R. Tuttle, Thorsten Vetter, Angela Yee Moon Wang, Faiez Zannad

**Affiliations:** 1 Medical Research Council Population Health Research Unit, Nuffield Department of Population Health, University of Oxford, Oxford, UK; 2 Clinical Trial Service Unit and Epidemiological Studies Unit (CTSU), Nuffield Department of Population Health, University of Oxford, Oxford, UK; 3 Department of Epidemiology, John Hopkins University, Baltimore, Maryland, USA; 4 University of British Columbia, Vancouver, British Columbia, Canada; 5 The George Institute for Global Health, University of Sydney, Sydney, New South Wales, Australia; 6 Cardiovascular Division, Brigham and Women’s Hospital, Harvard Medical School, Boston, Massachusetts, USA; 7 Steno Diabetes Centre Copenhagen, Copenhagen, Denmark; 8 University of Copenhagen, Copenhagen, Denmark; 9 Population Health Research Institute, Hamilton Health Sciences/McMaster University, Hamilton, Ontario, Canada; 10 Würzburg University Clinic, Würzburg, Germany; 11 Centre for Nephrology, University College London, London, UK; 12 Selzman Institute for Kidney Health, Section of Nephrology, Department of Medicine, Baylor College of Medicine, Houston, Texas, USA; 13 British Heart Foundation Cardiovascular Research Centre, University of Glasgow, Glasgow, UK

**Keywords:** kidney disease, randomized clinical trials, trial conduct, trial design

## Abstract

Despite the high costs of treatment of people with kidney disease and associated comorbid conditions, the amount of reliable information available to guide the care of such patients is very limited. Some treatments have been assessed in randomized trials, but most such trials have been too small to detect treatment effects of a magnitude that would be realistic to achieve with a single intervention. Therefore, KDIGO convened an international, multidisciplinary controversies conference titled “Challenges in the Conduct of Clinical Trials in Nephrology” to identify the key barriers to conducting trials in patients with kidney disease. The conference began with plenary talks focusing on the key areas of discussion that included appropriate trial design (covering identification and evaluation of kidney and nonkidney disease outcomes) and sensible trial execution (with particular emphasis on streamlining both design and conduct). Break out group discussions followed in which the key areas of agreement and remaining controversy were identified. Here we summarize the main findings from the conference and set out a range of potential solutions. If followed, these solutions could ensure future trials among people with kidney disease are sufficiently robust to provide reliable answers and are not constrained by inappropriate complexities in design or conduct.

Chronic kidney disease (CKD) affects ∼10% of adults in high-income countries,^1^, ^2^ and its treatment is burdensome and costly, accounting for substantial proportions of health budgets.^3^, ^4^ It is now recognized as an important cause of death, particularly in low- and middle-income countries where diabetes is becoming common and resources for treatment are limited.^5^, ^6^ Other conditions affecting the kidney, such as acute kidney injury^7^, ^8^ and kidney stone disease,^9^ also contribute a substantial burden of morbidity and mortality. Despite the high individual patient and societal burden of kidney diseases, the amount of reliable information available to guide kidney patient care is very limited.^10^ Although some treatments have been assessed in randomized trials, most of these have been too small to detect treatment effects of a magnitude that can realistically be achieved with a single intervention (e.g., reductions of 15%–20% in major outcomes such as death or disability). Experience to date indicates that conducting trials in patients with kidney disease on the sort of scale that has led to major therapeutic advances in other specialties (e.g., oncology, diabetes, and cardiology^11^) is challenging.

### Conference structure

In order to discuss the most significant barriers to conducting trials in patients with kidney disease and to propose potential solutions, KDIGO (Kidney Disease: Improving Global Outcomes) convened an international multidisciplinary Controversies Conference in Paris, France, titled “Challenges in the Conduct of Clinical Trials in Nephrology” in September 2016. The meeting began with plenary talks that aimed to identify the key themes for discussion related to trial design (including the specific topics of how to measure kidney and nonkidney outcomes) and trial execution before 4 breakout groups considered the key issues in detail. After each of the breakout sessions, ongoing deliberations were reported and discussed in plenary sessions. This paper synthesizes the main areas of discussion, agreement, and remaining controversies addressed at the conference. The conference agenda and selected presentations from the meeting are available on the KDIGO website (http://kdigo.org/conferences/clinical-trials/).

The discussion was based on the general principle that, in order to conduct a successful randomized trial, there are 4 main requirements: randomization of a sufficient number of patients (to ensure sufficient numbers of outcomes); assurance of adherence to the allocated treatment; reliable ascertainment of relevant study outcomes; and appropriate statistical analysis.^12^ These requirements can be met through a combination of sound trial design and efficient conduct. Much of the discussion at the conference centered on the concept of “streamlined” trial design, which focuses on the main determinants of trial quality and avoids unnecessary elements of design or conduct that increase cost and complexity.^13^

Trial design (analogous to experimental design) and trial conduct were considered separately. However, it should be noted that many aspects of a trial’s design influence its conduct and can therefore be decisive in determining whether a trial is successful. Because of their large scope, discussions about trial design were organized into 3 breakout groups that considered the design of trials in kidney disease, how to measure different kidney-specific outcomes, and assessment of other outcomes. The fourth breakout group focused on optimizing trial conduct. Table 1 brings together key findings from the breakout groups with regard to challenges in the design and conduct of randomized trials in patients with kidney disease. More complete details are provided in the following.

**Table 1:** Objectives in designing and conducting randomized trials and issues in the context of kidney disease

Trial objectives	Elements that help to achieve trial objectives		Difficulties in kidney disease
	Study design	Study procedures	
Answer an important question reliably	Differences between treatment(s) must be important to patients Sample size: needs to be determined by realistic assumptions (treatment effects, rates of adherence [drop-out/drop-in], and contemporary event rates), and is generally best determined by a fixed number of primary outcomes (i.e., event driven) Study duration: sufficient time is required for study treatments to exert benefit (or for any known hazards to emerge) Outcome selection: • Relevant to patients and must be measurable without undue burden on them • Sensitive to the main benefits and hazards of the trial treatment(s)		Many treatments are already in use despite a lack of reliable evidence of safety or efficacy. Nephrologists may be reluctant to compare such treatments with placebo. Because of the difficulties of identifying large numbers of eligible patients (especially in rare diseases), trialists may: • assume unrealistically large relative risk reductions (sometimes based on implausible results of systematic reviews or nonrandomized studies) • fail to allow for often substantial nonadherence, which severely diminishes statistical power Many kidney disease trials have not measured suitable outcomes. Common problems include: • Lack of relevance to patients, prescribers, and payers • Lack of consistency with outcomes in other pivotal trials • Use of total mortality, either alone or as a component of a composite primary outcome, resulting in a lack of statistical power
Effective recruitment	Population selection: • Trials should be relevant to a wide range of patients who might in the future be treated with the study intervention • Avoid unnecessary exclusions	Availability of large numbers of potentially eligible patients from routine databases, with prescreening (if feasible) Pilot study experience	Trials of patients with kidney disease often exclude large proportions of patients, resulting in both difficulty with recruitment and a lack of generalizability
Achieving good adherence	Exclude participants likely to drop out or drop in at screening or before randomization	Use of a run-in Procedures that are not onerous for trial participants Allow flexibility in determining nontrial treatments	Patients with kidney disease have a high burden of medication and intervention Nephrologists can become certain about benefit or harm before treatments are adequately tested
Complete recording of outcomes	Outcome definition does not require complex procedures or difficulties for patients Simple case report forms recording outcomes	Multimodal sources of patient data (patients, family members, primary care physicians) Maintaining contact with patients who no longer wish to take study treatment or attend clinics (e.g., by telephone follow-up) Use of registry data and electronic health care records	
Unbiased analysis	Statistical analysis plan, including: • Intention-to-treat analyses as primary • Limited number of subgroup analyses and only when hypotheses can be stated in advance • Clear demarcation of primary, secondary, and exploratory analyses		Underpowered trials and low adherence resulting in “negative” results for the primary outcome have previously led to inappropriate emphasis on underpowered subgroup analyses and potentially biased on-treatment analyses

### Optimizing trial design

#### General trial design considerations

The discussions identified important trial design principles that are generic but inconsistently applied in trials among patients with kidney disease, as well as issues that are specific to such trials.

#### Formulating the trial question

A key first step when planning a trial is to formulate the trial question. This should be both clinically relevant (i.e., addressing a major area of clinical uncertainty) and relevant to patients (i.e., aiming to avoid an outcome or condition that patients themselves consider significant). Patients should be involved in discussions when planning trials.

An important underlying principle in trials testing superiority of an intervention is that the aim should always be to compare treatment arms that differ substantially in their expected effects on the primary outcome and to preserve this separation for the duration of the trial. The likely magnitude of this difference can sometimes be usefully tracked through assessing differences in a surrogate variable between randomized treatment groups (e.g., plasma low-density lipoprotein cholesterol in the SHARP trial^14^), provided that blinding at an individual participant level is strictly enforced. Failure to maintain adequate adherence to study treatment leads to a loss of separation between groups and loss of statistical power. This may be a particular problem in trials among patients with CKD who are typically receiving multiple treatments, and are often required to attend clinics frequently, and are particularly prone to drug toxicity and intercurrent illnesses that may require the trial treatment to be modified (as observed in the EVOLVE trial in which study drug discontinuation was much higher than anticipated).^15^, ^16^

#### Selecting a suitable trial population

As a general principle, eligibility criteria should be practical and broad. This ensures a widely generalizable result and facilitates efficient recruitment. Inclusion criteria should identify a suitable population of patients who are likely to have the type of outcome that the treatment is anticipated to prevent. Guideline committees may help to maximize the size of eligible populations by highlighting areas of uncertainty, by avoiding making recommendations where evidence is weak, and by stating where placebo-controlled trials are needed.

Exclusion criteria should be constructed that exclude patients who have a definite indication or contraindication for at least 1 of the study treatments, who are likely to be nonadherent to the trial protocol, or who are not expected to survive for the duration of the trial. Therefore, patients who would have obvious safety issues (from relevant comorbidity) or who are at high risk of a potential pharmacologic interaction related to the intervention should generally be excluded (although this might not always be necessary if a Data and Safety Monitoring Board is charged with monitoring safety in specific patient groups). An important group to exclude is those who are likely to stop allocated treatment because of intolerance or nonadherence (“drop-out”) or who are likely to start the study drug (or a drug with a similar mode of action) outside of the trial (“drop-in”). It may be possible to identify patients who are likely to be nonadherent during a prerandomization “run-in” period so that they can be withdrawn before being randomized. This helps to preserve study power.^17^ Run-in periods may be particularly beneficial in trials of dialysis patients, a complex group for which maintaining adherence to generally well-tolerated medications can be difficult.^14^, ^15^

#### Calculating an appropriate sample size

Kidney disease has a wide variety of causes, and many different pathophysiologic mechanisms are responsible for disease initiation and progression, as well as for its complications. This makes it unlikely that a single treatment would have a large relative effect on major outcomes (particularly on outcomes such as death or progression to end-stage kidney disease). In determining sample size, therefore, particularly for large Phase 3 outcome trials, it is important to avoid overly optimistic assumptions about treatment effect size, even when there are apparently large effects on disease biomarkers. In practice, this means that relative risk reductions in major outcomes of more than ∼20% are unlikely to be observed. Nonetheless, in high-risk patients such as those with kidney disease, small relative risk reductions may still correspond to large reductions in absolute risk and therefore be clinically and economically worthwhile.

It is inappropriate to estimate relative risk reductions (for major outcomes) from systematic reviews composed solely of small randomized trials; such trials can only achieve statistically significant results (and hence publication) if—by the play of chance—their effect estimates are larger than the truth, which means that such reviews will tend to yield inflated effect size estimates.^18^ When the evidence from previous small trials is limited in this way, a trial that is capable of detecting a realistically moderate effect (e.g., a 15%–20% reduction in a major outcome) is more rational than one that aims to detect an effect estimated in a systematic review. When designing kidney trials, there may be considerable uncertainty when estimating event rates owing to a lack of contemporary data from population-based studies.^19^ In addition, event rates derived from population-based registries may not reflect those of an enrolled trial population. The potential for high levels of nonadherence to study treatment, which can have a particularly detrimental effect on study power, should also be considered. Adequate drug exposure and study power may therefore be achieved by planning on a predefined total number of primary outcomes and a minimum duration of follow-up in a study population of approximately the correct size. A Data and Safety Monitoring Board can advise on early termination of a trial if convincing evidence of harm or efficacy emerges before trial completion or if there are other reasons (e.g., irremediable failure to recruit or futility) that make it inappropriate to continue.

#### Statistical analysis

The principles of statistical analysis for randomized trials are well documented and should be applied in trials among patients with kidney disease.^12^ For example, intention-to-treat analyses should be specified for the primary analysis. Several particular problems are commonly encountered. First, where sample sizes are limited, there has been a temptation to overinterpret subgroup findings or where nonadherence is a problem, to conduct potentially biased on-treatment analyses. In superiority trials, such analyses should be considered exploratory rather than confirmatory, except where the analysis plan provides a clear justification for them before database lock and consideration has been given to the number of comparisons being made.

In reporting the results of a clinical trial, the primary outcome should be emphasized, even if the results do not support the intervention. However, the full interpretation of a trial’s results should relate to the totality of evidence, meaning the primary outcome plus secondary and safety outcomes.^20^ If evidence is indeed inconclusive, this is an acceptable and valuable conclusion, and further randomized trials may still be useful if uncertainty remains.

### Selection of outcomes for assessing treatment effects

#### Measurement of kidney-specific outcomes

The choice of outcome measures in trials designed to assess kidney disease status (i.e., function, damage, or disease activity) should depend on the disease setting and phase of clinical development. Consideration should be given both to the stage of the disease and how rapidly it is progressing. Table 2 provides a matrix of general approaches to and strategies for selecting endpoints. The measurement of activity of disease and kidney structure, if available, may add useful information to measures of kidney function. Disease-specific markers (or outcomes) that reflect the underlying pathophysiology or molecular pathways of disease may be helpful in the setting of primary kidney diseases such as systemic lupus erythematosus, IgA nephropathy, and polycystic kidney disease. However, in settings where progression of kidney disease is a multifactorial process, such as with hypertension or diabetes mellitus, a kidney-specific outcome may be best assessed using the estimated glomerular filtration rate (eGFR).^21^

**Table 2:** Suggested outcomes in measuring kidney disease status in randomized trials

CKD stage	Progression of CKD	
	Slow	Rapid^a^
Early stage: CKD G1-G3a (eGFR ≥45 ml/min per 1.73 m^2^)	• Slope of mGFR or eGFR or • Surrogate outcome^b^ or • Combinations of outcomes	30%−40% decline in eGFR using repeat measurements to rule out transient acute effects^c^
Late stage: CKD G3b-G5 (eGFR <45 ml/min per 1.73 m^2^)	End-stage kidney disease or 30%−40% decline in eGFR^c^	End-stage kidney disease or doubling of serum creatinine level (or 40%–57% decline in eGFR)^c^

CKD, chronic kidney disease; eGFR, estimated glomerular filtration rate; mGFR, measured glomerular filtration rate.

a For example, in patients with macroalbuminuria.

b Surrogates may include measures of activity of disease (e.g., in lupus nephritis) or kidney structure (e.g., in adult polycystic kidney disease).

c The added value of eGFRs outside the routine study visit schedule has not yet been demonstrated and they may be unnecessary.

When outcome measures reflect the underlying pathophysiology of a disease, the effects of an intervention may be large enough to detect reliably in small to medium sized trials. Therefore, surrogate outcomes are suited to smaller Phase 2 clinical trials used to establish proof-of-concept, optimal drug dose, and information on tolerability. Markers of glomerular or tubular damage, inflammation, fibrosis, etc., or a combination of markers may be considered at this phase. A demonstrable difference in the average eGFR (or change in the eGFR) between treatment groups may also be possible in small- to medium-size trials.

In larger Phase 3 trials, it may also occasionally be appropriate for outcome measures in support of the primary outcome to include measures of structural damage or disease-specific markers. Acceptable surrogates include measuring total kidney volume in autosomal dominant polycystic kidney disease.^22^, ^23^ However, such trials must be sufficiently large to also provide reliable information on the safety of the intervention. Changes in eGFR over time may remain a more practical and acceptable method for assessing progression of kidney disease in many trials (Table 2).^24^

Directly measured GFR may occasionally be necessary if a treatment might influence variables used to measure eGFR through mechanisms other than effects on glomerular filtration (e.g., muscle mass changes or tubular secretion of creatinine). In the pediatric population, most clinical research is conducted in the setting of rare diseases, which, by definition, have small disease populations. Even so, studies in pediatric populations are subject to the same principles for evaluating progression of CKD as in adults. However, using creatinine as a marker of GFR is problematic in children younger than 2 years of age because creatinine levels rise rapidly during infancy. The European Medicines Agency provides guidance on extrapolating efficacy and safety data from adults to children to inform pediatric investigation plans.^25^

An important current issue is whether change in albuminuria is acceptable as a surrogate marker of CKD progression.^26^ In the context of nephrotic syndrome, large changes in albuminuria are an acceptable marker of kidney disease activity. Albuminuria may also be appropriate in the setting of structural damage, but it may not be in the setting of hemodynamic dysfunction or acute reversible disease. Albuminuria may also be an appropriate surrogate if there is evidence that the effects of treatment are durable. Possible endpoints for evaluating treatments include prevention of incident macroalbuminuria, remission from macroalbuminuria to normoalbuminuria, and a predetermined decrease, such as a set quantitative change. More data are needed to better understand whether and how changes in albuminuria correspond to disease progression and how changes can be meaningfully applied to designing trial endpoints.^27^ The US National Kidney Foundation, the US Food and Drug Administration, and the European Medicines Agency will convene a meeting on March 15 to 16, 2018, to discuss these issues.^28^

#### Measurement of comorbidity and mortality

Outcomes that measure aspects of disease status other than kidney structure or function may be disease-specific morbidity or mortality and can include assessments of quality of life or everyday functioning or economic impact. Outcomes should capture the expected, plausible treatment effects (benefits and harms), be relevant to patients and health care providers, and be appropriate for the phase of clinical development. Excess burden to patients, their families, health care providers, and research staff should be avoided. When available, measurement instruments that have operating characteristics known to be within acceptable limits should be used.

In determining outcome measures, patient and caregiver perspectives should be sought and considered for all clinical trials in nephrology. Standard definitions for key core outcomes identified as being important to patients on dialysis or with kidney transplants are being developed by the Standardised Outcomes in Nephrology initiative.^29^, ^30^ For example, outcomes that the Standardised Outcomes in Nephrology initiative has identified to be important to patients on hemodialysis include mortality, functioning of vascular access, and cardiovascular disease, but also include symptoms such as fatigue, pruritus, cognitive function, and functional limitations. The US Food and Drug Administration has provided specific guidance on how Patient Reported Outcomes, which include assessments of quality of life, can be developed and validated to assess these symptoms to support labeling claims.^31^ Examples for which streamlined Patient Reported Outcomes may be particularly important include trials of treatments for anemia (alongside outcomes related to clinical safety and efficacy).

Composite outcomes should combine components that make sense for the specific intervention, patient population, and disease state and should be of approximately comparable clinical importance. All-cause mortality is rarely an appropriate primary outcome in kidney trials because it is neither sensitive to any real effects on particular causes of death nor generalizable to different types of patients. It is preferable to create composite outcomes comprising related events that are all likely to be influenced favorably by treatment (and assess the effects on safety outcomes separately). If kidney and cardiovascular outcomes are to be combined in a single composite primary outcome, it is important to ensure that sufficient information will be available on both disease components to be able to guide treatment decisions. Similarly, the use of co-primary endpoints (for which an effect has only to be demonstrated on one of them) is not generally appropriate unless each is in some way relevant to patients and analyses of all such endpoints are adequately powered.

When available and appropriate for the particular clinical context under study, standardized disease outcome definitions that are feasible to apply at scale should be considered. However, if needed, new definitions should be considered in special situations such as heart failure in the context of dialysis. When a composite outcome is used, it is important to assess the effects of treatment on its components and related outcomes. Analyses of events that recur (e.g., vascular access procedures or hospitalization for heart failure) may be important to patients and payers, and appropriate statistical methodology to analyze recurrent events is available.^32^ Where continuous measures are used, clinically important differences should be defined and justified.

Instruments assessing health-related quality of life can assess the economic impact of a treatment to inform payers. Application of such instruments should follow the same principles as those for other patient populations without kidney disease, and appropriately streamlined methods for gathering such data should be included in trial designs.

### Optimizing trial conduct

Increasing the number of large, important, and relevant clinical trials will require a culture shift within the nephrology community. A multipronged approach is required to improve study conduct and help all stakeholders to better understand the ways in which high-quality clinical trials improve patient care. One practical solution for streamlining the conduct of trials is integrating research processes and procedures into routine care (as has been done so successfully in oncology, diabetes, and cardiology^33^), and success in this depends on improved community awareness.

Inefficiencies in clinical trial conduct jeopardize the ability to address important clinical questions and are a disservice to trial participants. Table 3 lists potential strategies to improve the efficiency and effectiveness of clinical trials in nephrology and in other disciplines. Such strategies would be expected to enhance the rights of participants (e.g., by ensuring that consent procedures provide information in an accessible form) as well as their safety and well-being (e.g., by minimizing the requirements for study visits and invasive tests or by more effective methods of pharmacovigilance). This work builds on the Quality by Design approaches developed by the Clinical Trials Transformation Initiative.^34^, ^35^

**Table 3:** Strategies for minimizing issues that have a meaningful impact on the rights, safety, and well-being of trial participants or on the reliability of the trial conclusions (which will influence the care of future patients)

1. Facilitating efficient and rapid recruitment
2. Streamlining the process of high-quality data collection (by assessing a limited number of critical data elements)
3. Maximizing adherence to study treatment and minimizing loss to follow-up
4. Improving the efficiency and appropriateness of trial monitoring (including using risk-based central statistical processes)
5. Rationalizing safety monitoring and pharmacovigilance activity (with more focus on the review of randomized comparisons of aggregated data by the unblinded Data and Safety Monitoring Boards)
6. Tailoring adjudication methods to focus on those events in which adjudication may materially influence interpretation of the results

#### Recruitment

Increasing participation in clinical trials is a major goal and requires a range of strategies (Table 4). Patients and clinicians should be made aware of the value of research participation and the importance of randomization. This could be done through education via videos, webinars, targeted advertising strategies, or peer group discussions. Educational efforts, both formal and informal, need to be dedicated, consistent, and constant. Patient advocacy groups for rare diseases have been successful at engaging patients and providers about the meaning and value of research and are a potential resource for ideas and collaboration.

**Table 4:** Strategies to improve recruitment into kidney disease trials

Education
● Demonstrate to the kidney health community the value of research participation using visual media (i.e., social media, charity/patient advocacy group websites, webinars) and peer group discussion
● Provide nephrologists with examples of the importance of uncertainty
● Develop annual kidney clinical trials education for the community (providers and patients)
• Improve knowledge of the principles of clinical trial design and conduct
• Identify global and local barriers to conducting quality trials
• Share successes/tools
● Increase trial awareness through local advertising and patient advocacy groups
● Develop systems for peer review of protocols for new trialists
Improve information on potential trials
● Within individual health care systems and clinic settings:
• Create a readily accessible repository of current and planned trials
• Create or use existing electronic health care records or registries to identify eligible patients (particularly for rare diseases)
Improve trial infrastructure
● Widen the type of health care services participating in trials
Incentivize trial participation
● Acknowledge clinical research activities (e.g., using continuing medical education credits, “awards”)
● Nationally audit trial participation as a marker of quality of care
● Payers to reward randomization
Make randomization easy
● Simplify consent procedures
● Integrate trial systems into the electronic systems used in routine practice
Cross-collaborate with other specialties
● Develop trials with diabetologists, cardiologists, and other specialists

Processes for informing patients and health care team members about specific clinical trials should be systemically embedded in health care communities. At individual centers, repositories of current and planned studies should be accessible via various internet portals, and information about studies should be displayed in the waiting areas and offices of medical facilities. For recruiting into specific trials, it may be helpful to institute a process of “prescreening” whereby research coordinators develop lists of potentially eligible patients and, where permissible, provide those patients with information about the trial and “preconsent” them. This then enables recruitment to proceed rapidly once the trial receives full ethical and regulatory approval (and also helps to identify sites that will not have sufficient patients to contribute). Electronic health records may provide an opportunity to identify large numbers of potential participants who may be eligible and should be invited. Widespread invitation ensures that patients are empowered to decide whether they want to participate in a trial rather than waiting for their doctor to hand-select them.

Research champions within countries and regions should be recognized and identifiable. Clinical research organizations, academic research organizations, and networks of trialists should be encouraged to share information about the enrollment performance of individual study sites. This could speed completion of enrollment and reduce wasting of resources on poor performing sites. Excellence in research should also be recognized by national audits and by payers, with rewards given both for randomization and success in achieving quality data and completeness of follow-up.

#### Data collection

The amount and type of data collected in a clinical trial affect the ability to recruit patients, follow their progress, and complete the trial. Inefficiencies in data collection increase trial costs, labor, and burden of participation (for both participants and the research team). To date, trials in patients with kidney disease have tended to collect too many data fields, most of which do not contribute to answering the main clinical question and lead to unnecessary complexity and difficulty in recruiting.^34^, ^35^ Researchers should identify small core datasets required for each study, minimize the frequency of measurements, and simplify the collection of data. The specific processes developed for an individual study will vary depending on the study’s purpose, type of intervention, available resources, and stage of development.

Well-established national dialysis and transplant registries in many countries provide an opportunity to streamline data collection in trials. Focused kidney-specific templates that use consistent terminology, definitions, and sets of variables could be of value if these templates were integrated into registries or electronic health records.

Depending on the circumstances, expensive central laboratory analyses may not be essential where an outcome is a measure of difference in a biomarker between randomized arms. Variation in calibration for a biomarker between laboratories has little impact on the magnitude of *differences* between randomized groups. For example, in the SHARP trial, analyses of differences in routine plasma (or serum) creatinine measured every 6 months at local hospital laboratories allowed low-cost, but reliable, assessment of the effects of ezetimibe/simvastatin on the progression of CKD.^14^ When using local laboratories, however, it is important to know their reporting units, reference ranges, and analytic methods to ensure that summary analyses are meaningful.

There is increasing evidence that verification of clinical outcome data (usually referred to as outcome adjudication) may have little effect on the relative risk reductions reported by trials and that this process could also be streamlined in certain situations.^36^

#### Maximizing adherence to treatment and follow-up procedures

Approaches such as using “run-in” periods to identify patients who are unlikely to adhere to study treatment and/or attend clinics,^17^ minimizing unnecessary data collection, limiting excessive numbers of study visits (perhaps by arranging follow-up by telephone or electronic health records where possible), and expediting in-person visits (e.g., by avoiding lengthy waits in the hospital pharmacy) may all help maintain adherence and follow-up (Table 1). Adherence should be monitored centrally, and each study treatment “dropout” should prompt investigation into the reason and discussion as to whether study treatment can be restarted.

In clinical studies, the term *withdrawal of consent* is problematic because of its lack of specificity and because it is frequently confused with a participant’s wish to stop study treatment or not undergo a certain trial procedure. Specific levels of withdrawal from the protocol-specified follow-up, which range from a patient not attending clinic visits but perhaps agreeing to clinical note review to an absolute withdrawal with no further data being provided, should be embedded in case report forms. To better capture follow-up data for patients who are no longer participating in a study, patients could be asked to agree that electronic health records can be accessed. This would allow for capturing information about their outcomes, adverse events, and concomitant medications during and after study participation.^37^ Engaging general practitioners and primary care providers could also help in establishing streamlined methods for complete study participant follow-up.

#### Trial monitoring

Trial monitoring can be time-consuming and resource intensive, and therefore simplifying these processes can have a profound impact on reducing the burden of the trial conduct. When using direct electronic data entry, processes for trial monitoring and source data verification can become much more efficient.^38^ For example, risk-based approaches to monitoring (focusing on those data that are critical to trial quality) and central statistical monitoring (using study data to identify unusual patterns of performance) can reduce and prioritize site visits.

Safety reporting should be tailored for each trial protocol. In early phase development, rigorous detailed adverse event ascertainment is necessary, but this level of event recording may not be necessary when the safety profile of a treatment is well-known. During protocol development, regulators such as the US Food and Drug Administration and European Medicines Agency can advise on which specific adverse events need to be collected and which do not. Regulators can also advise on the level of information that needs to be collected. Clinical narratives are burdensome, may reduce trial participation, and should be focused only on those serious adverse events where such data may be informative, such as suspected unexpected serious adverse reactions.^39^ Reliable review of safety during a trial is best achieved by examining randomized comparisons of aggregated data by the unblinded Data and Safety Monitoring Boards.

### Conclusions

The lack of adequately powered randomized trials in nephrology has led to a problematic imbalance between the clinical need of patients with kidney disease and the amount of reliable evidence to inform practice. This KDIGO conference highlighted some of the key challenges faced by those trying to perform large trials. These include a lack of uncertainty among nephrologists who have often adopted treatments before adequate evidence of efficacy or safety is available, smaller treatment effects than were predicted from effects on surrogate biomarkers, inappropriate selection of outcomes including those with little relevance to patients or unlikely to be affected by treatment (e.g., all-cause mortality), difficulty in identifying large numbers of eligible patients, high levels of nonadherence to study treatment by overburdened patients, and overcomplicated trial conduct (Table 1). Adoption of the approaches outlined in this report has the potential to dramatically improve the quality of clinical trials in nephrology and substantially enhance the evidence base for the safe and effective treatment of patients with kidney disease.

## Disclosure

This conference was sponsored by KDIGO and was in part supported by unrestricted educational grants from Abbvie, Achillion, Akebia Therapeutics, Alexion, Amgen, AstraZeneca, Bayer HealthCare, Fresenius Medical Care, KBP Biosciences, Keryx Biopharmaceuticals, Merck, Omeros, Relypsa, Roche, and Vifor Fresenius Medical Care – Renal Pharma.

## Appendix

**Other conference participants**

Ali Abu-Alfa, Lebanon; Patrick Archdeacon, United States; Geoffrey A. Block, United States; Fergus J. Caskey, United Kingdom; Alfred K. Cheung, United States; Bruce Cooper, Australia; Jonathan C. Craig, Australia; Laura M. Dember, United States; Garabed Eknoyan, United States; Ron T. Gansevoort, The Netherlands; John S. Gill, Canada; Barbara Gillespie, United States; Tom Greene, United States; David C. Harris, Australia; Richard Haynes, United Kingdom; Brenda R. Hemmelgarn, Canada; Charles A. Herzog, United States; Thomas F. Hiemstra, United Kingdom; Lesley A. Inker, United States; Meg J. Jardine, Australia; Vivekanand Jha, India; Lixin Jiang, China; Kirsten L. Johansen, United States; Reshma Kewalramani, United States; Hiddo J. Lambers Heerspink, The Netherlands; Martin Lefkowitz, United States; Charmaine E. Lok, Canada; Fiona Loud, United Kingdom; Romaldas Mačiulaitis, United Kingdom; Dugan W. Maddux, United States; Franklin W. Maddux, United States; Magdalena Madero, Mexico; Segundo Mariz, United Kingdom; Michael Mauer, United States; Joseph V. Nally, Jr., United States; Masaomi Nangaku, Japan; Ikechi G. Okpechi, South Africa; Patrick S. Parfrey, Canada; Roberto Pecoits-Filho, Brazil; Brian J. G. Pereira, United States; Michael V. Rocco, United States; Patrick Rossignol, France; Franz Schaefer, Germany; Francesca Tentori, United States; Aliza Thompson, United States; Marcello Tonelli, Canada; Allison Tong, Australia; Robert D. Toto, United States; Katherine R. Tuttle, United States; Thorsten Vetter, United Kingdom; Angela Yee Moon Wang, Hong Kong; Faiez Zannad, France.
